# Cost-utility analysis of a one-time supervisor telephone contact at 6-weeks post-partum to prevent extended sick leave following maternity leave in The Netherlands: results of an economic evaluation alongside a randomized controlled trial

**DOI:** 10.1186/1471-2458-11-57

**Published:** 2011-01-27

**Authors:** Kimi Uegaki, Suzanne GM Stomp-van den Berg, Martine C de Bruijne, Mireille NM van Poppel, Martijn W Heymans, Willem van Mechelen, Maurits W van Tulder

**Affiliations:** 1 Health Technology Assessment Unit, EMGO Institute for Health and Care Research, VU University Medical Center, Amsterdam, The Netherlands; 2 Department of Public and Occupational Health, EMGO Institute for Health and Care Research, VU University Medical Center, Amsterdam, The Netherlands; 3 Body@Work, Research Center Physical Activity, Work and Health, TNO-VUmc, Amsterdam, The Netherlands; 4 EMGO Institute for Health and Care Research and Department of Clinical Epidemiology & Biostatistics, VU University Medical Center, Amsterdam, The Netherlands; 5 Institute of Health Sciences, Faculty of Earth and Life Sciences, VU University, Amsterdam, The Netherlands

## Abstract

**Background:**

Working women of childbearing age are a vital part of the population. Following childbirth, this group of women can experience a myriad of physical and mental health problems that can interfere with their ability to work. Currently, there is little known about cost-effective post-partum interventions to prevent work disability. The purpose of the study was to evaluate whether supervisor telephone contact (STC) during maternity leave is cost-effective from a societal perspective in reducing sick leave and improving quality-adjusted life years (QALYs) compared to common practice (CP).

**Methods:**

We conducted an economic evaluation alongside a randomized controlled trial. QALYs were measured by the EuroQol 5-D, and sick leave and presenteeism by the Health and work Performance Questionnaire. Resource use was collected by questionnaires. Data were analysed according to intention-to-treat. Missing data were imputed via multiple imputation. Uncertainty was estimated by 95% confidence intervals, cost-utility planes and curves, and sensitivity analyses.

**Results:**

541 working women from 15 companies participated. Response rates were above 85% at each measurement moment. At the end of the follow-up, no statistically significant between-group differences in QALYs, mean hours of sick leave or presenteeism or costs were observed. STC was found to be less effective and more costly. For willingness-to-pay levels from €0 through €50,000, the probability that STC was cost-effective compared to CP was 0.2. Overall resource use was low. Mean total costs were €3678 (95% CI: 3386; 3951). Productivity loss costs represented 37% of the total costs and of these costs, 48% was attributable to sick leave and 52% to work presenteeism. The cost analysis from a company's perspective indicated that there was a net cost associated with the STC intervention.

**Conclusions:**

STC was not cost-effective compared to common practice for a healthy population of working mothers; therefore, implementation is not indicated. The cost-utility of STC for working mothers with more severe post-partum health problems, however, needs to be investigated. Work presenteeism accounted for half of the total productivity loss and warrants attention in future studies.

**Trial registration number:**

ISRCTN: ISRCTN73119486

## Background

Worldwide working women of childbearing age are a vital part of the population. Following childbirth, this group of women can experience a myriad of physical and mental health problems that can interfere with their ability to work [1-3]. Yet, discussions surrounding postpartum return-to-work and maternal health are infrequent [4]. Sick leave is common. Van Beukering [2] reported that - following maternity leave - 29% of Dutch working women took two or more weeks of sick leave. In 55% of these cases, sick leave periods exceeded 12 weeks. Furthermore, Cuelenaere et al. [5] reported that the annual percentage of women with postpartum sick leave who qualified for work disability benefits was higher than the rest of the working population: 5% versus 1.5%. These physical and mental health problems may also interfere with a woman's ability to work once she has returned to the workplace, that is, result in work presenteeism [6]. While statistics on work presenteeism among women following childbirth are currently lacking, there is growing evidence of work presenteeism among workers with health problems such as back or other musculoskeletal pain, fatigue and depression [7,8]. Given the similarity with the types of health problems faced by women following childbirth, the existence of work presenteeism is plausible.

In practice, post-partum health care by the midwife, obstetrician/gynaecologist or general practitioner covers the first six weeks and is focused on the recovery of the women's reproductive system, not on physical or mental health barriers to return-to-work. In the Netherlands, maternity leave is 16 weeks long with full pay, and typically divided into 4-6 weeks before the delivery date and 10-12 weeks thereafter. Traditionally, Dutch working women have received little active support by occupational health physicians during the post-partum period [9], and occupational health case management during the 10-12 week period after childbirth remains poorly defined. Occupational health physicians are consulted when return-to-work at the end of maternity leave period has been postponed by two to eight weeks of sick leave. This can result in a situation of non-treatment and prolonged sick leave. The former is paired with a higher risk for chronicity and the latter is associated with a reduced probability of return-to-work. Currently, there is little known about cost-effective post-partum interventions that can help prevent work disability.

While the role of supervisors in facilitating return-to-work of employees on sick leave and case management in The Netherlands is legislated [10], the scope excludes involvement during maternity leave. Previous research has shown that supervisors can play a key role in facilitating return-to-work [11]. As such, an intervention in which supervisors make contact with workers during maternity leave was developed and evaluated with the hypothesis that this would reduce sick leave after childbirth [12]. The objective of this study was to determine whether supervisor telephone contact was cost-effective from a societal perspective in reducing post-partum sick leave and improving health-related quality of life compared to common practice. We also described the resource use, productivity loss and associated costs of this study population during the first year post-partum.

## Methods

### Study design

This was an economic evaluation alongside a randomized control trial [12]. We selected a societal perspective because in The Netherlands, current legislation divides the financial responsibility for sick leave benefits between social security (i.e. when sick leave is subsequent to maternity leave) and companies (i.e. when sick leave occurs after a period of return to work following maternity leave). Participants were followed for 52-weeks post-partum. The protocol was approved by the Medical Ethics Committee of the VU University Medical Centre, Amsterdam, The Netherlands. We obtained written informed consent from all participants.

### Participant recruitment

The sample size calculation was based on an expected 10% decrease in the number of women taking sick leave at the end of maternity leave, a power of 80% and an alpha-level of 0.05. A total of 550 pregnant working women were needed.

The recruitment procedure consisted of two steps: 1. recruitment of companies; and 2. recruitment of pregnant workers within these companies. For practical reasons, we aimed our recruitment at large companies with a predominantly female workforce. Based on reports of higher sick leave rates before and after maternity leave in the health care sector [2], health care providers (e.g. hospitals, home health and occupational health service companies) were over-sampled.

The recruitment of pregnant workers took place from January 1, 2004 through March 31, 2006. Within each participating company, the recruitment procedure was initiated by the human resource departments. When pregnant workers submitted requests for maternity leave, they received an information package about the study: letter of invitation, a study leaflet, two response cards, and a return envelope. If a woman returned a completed 'yes' response card, then researchers contacted her to verify eligibility, obtain informed consent and conduct the baseline measurement.

The inclusion criteria were: 1. aged 18 through 45 years; 2. employed a minimum of 12 hours per week; and 3. clear intention to return-to-work after maternity leave to the same employer for a minimum of 12 hours per week and for a minimum period of 6 months. The exclusion criteria were: 1. miscarriage; 2. delivery before 34 weeks; 3. a request for full work-disability benefits submitted; 4. receipt of full work-disability benefits; and 5. uncertainty or clear intention not to return the same employer after maternity leave.

### Randomization & blinding

In order to prevent contamination, randomization took place at the level of the supervisor. For each participating company, a randomization list was computer-generated by an independent statistician assuring concealment of treatment allocation. When participants were 35 weeks pregnant, supervisors were randomized in blocks of four where each block contained two intervention and two control group allocations. Blocks of four were chosen because of the uncertainty in how many employees of each company would participate and some companies had a small number of employees. Supervisors and participants in the control group and data entry assistants were blinded to group allocation. Blinding during the data analysis was guaranteed by means of coded patient, supervisor and company data.

### The interventions

#### Supervisor telephone contact (STC)

The aim of the STC intervention was to prevent prolonged non-treatment of health problems that could delay return-to-work following the end of maternity leave by instigating the involvement of occupational health services 6-12 weeks earlier than in the usual situation. At 6-weeks post-partum, supervisors contacted their employees to conduct a standardized interview in order to identify health problems that may be barriers to RTW after the official end of maternity leave. If such health problems were identified, the support of the occupational health services was offered. This telephone interview was in addition to the usual congratulatory telephone calls, cards and visits. Supervisors received written and oral instruction about their role as case managers. At 6-weeks post-partum, respective supervisors received e-mail notification to carry out this intervention.

#### Common practice (CP)

In CP, there was no structured contact by supervisors to address health barriers to return-to-work during the maternity leave period. Health problems and delays in return-to-work may be discussed ad hoc during the maternity leave or for the first time at the end of the maternity leave period, at which time occupational health services may be offered. Congratulatory telephone calls, cards and visits took place as usual.

### Data collection and valuation

We collected cost data related to the health care sector, other sector and patient/family resource use using questionnaires at 6-, 12-, 18-, 24- and 52-weeks post-partum. With the exception of the 52-week post-partum measurement moment, the recall period for questionnaires was six weeks; at 52-weeks, the recall period was four weeks. With regards to productivity loss, we collected data on both sick leave and work presenteeism (i.e. decreased work performance due to a health problem) using the Health and work Performance Questionnaire (HPQ) [13] at 18-, 24- and 52-weeks post-partum. Data on the use of parental leave and the number of hours taken were also collected.

We collected extra cost data at 30-, 36-, 42- and 48-weeks post-partum if women reported consumed health care resources, reported sick leave or work presenteeism, or reported still not back to her 'old self' in the preceding measurement period. Otherwise, we did not send out extra questionnaires as we assumed the women were healthy and health care resources would not be consumed nor that productivity loss would occur.

We measured health-related quality of life using the EuroQol-5D [14] questionnaire at 6-, 12-, 24- and 52-weeks post-partum. Utilities were determined by Dutch tariffs as opposed to the original UK tariffs, given differences in health-related social preferences between the countries. The Dutch tariffs were developed using the time-trade off method where the anchors where 0 = death and 1 = full health [15,16]. Quality-adjusted life years (QALYs) were computed by multiplying the utilities by the time spent in the given health state, and then linearly interpolating the transitions between measured health states [17].

Wherever possible, we determined costs by multiplying the respective units of resource use by standard price weights according to the Dutch Manual for Costing [18]. If standard price weights were not available, we used tariffs or an average price according to providers or professional organization. We calculated medication costs using unit prices published by the Royal Dutch Society for Pharmacy [19]. Productivity loss costs due to sick leave were estimated using the Friction Cost Method (FCM) and the Human Capital Approach (HCA). For the FCM estimation, we used a friction period of 154 days and an elasticity of 0.8. Because follow-up was limited to one year, we did not discount the costs or outcomes [18]. Costs were reported in Euros and the index year was 2006.

### Data analysis

We analysed the data according to the intention-to-treat principle. There were 9 women who were lost-to-follow-up after randomization (STC = 5; CP = 4) for the following reasons: problems with the questionnaire (CP = 3), baby died (STC = 1), baby in hospital (STC = 1), no time (STC = 1; CP = 1), and no reason reported (STC = 2). Because cost and health-related quality of life data were not available for any of these subjects, we had to exclude them from the analyses. Twenty women became pregnant for a second time during the follow-up period; we included these women in the analysis, but treated the data following the time of second pregnancy as missing.

First, we analysed utilities and resource utilization rates using available data. Second, we determined mean units of resource use using complete cases. Descriptive analyses of utilities and resource use for each group and between-group differences were conducted in SPSS version 15.0. Third, we conducted the main cost, effect and cost-utility analyses after imputing partially missing data with a multiple imputation (MI) procedure based on Multivariate Imputation by Chained Equations (MICE) [20-23]. We defined the incremental cost-utility ratio (ICUR) as the difference in total costs (i.e. TC_FCM_, the sum of health care sector, other sector, patient/family, intervention and productivity loss costs estimated by the FCM) divided by the difference in QALYs. Baseline characteristics were comparable between the groups, therefore, we did not adjust our analyses for potential confounding. Also, we conducted conventional, uncorrected analyses because there was no effect of clustering at the supervisor or company levels. Finally, because there were no differences in effect between the STC and CP groups, we pooled resource use data from both groups to gain insight into resource use and costs during the first 52-weeks post-partum.

In the MI procedure, five imputed data sets were generated, each of which were analysed separately. We analysed the mean QALY differences parametrically [24]. The 95% confidence intervals around the mean cost differences and ICUR differences were obtained using an approximated bias-corrected (ABC) bootstrapping procedure with 1000 replications [25,26]. We applied Rubin's rules to obtain the pooled estimates of mean costs and QALYs, mean cost and QALY differences and 95% confidence intervals. To gain insight into the uncertainty around the pooled mean ICUR from the CUA, we plotted the bootstrapped cost-effect pairs in a cost-utility plane [27] and generated an acceptability curve [28]. The multiple imputation procedure and analyses of costs, effects and cost-utility were conducted in the software, R [29].

#### Sensitivity analysis

We conducted three sensitivity analyses to test the robustness of our main findings. First, we repeated the main CUA in which the total costs included productivity loss costs estimated via the HCA. Second, we conducted the CUA after excluding the 20 women who became pregnant for a second time during the follow-up period. Third, we repeated the CUA using only complete cases.

## Results

### Participants and data availability

**Figure 1:**
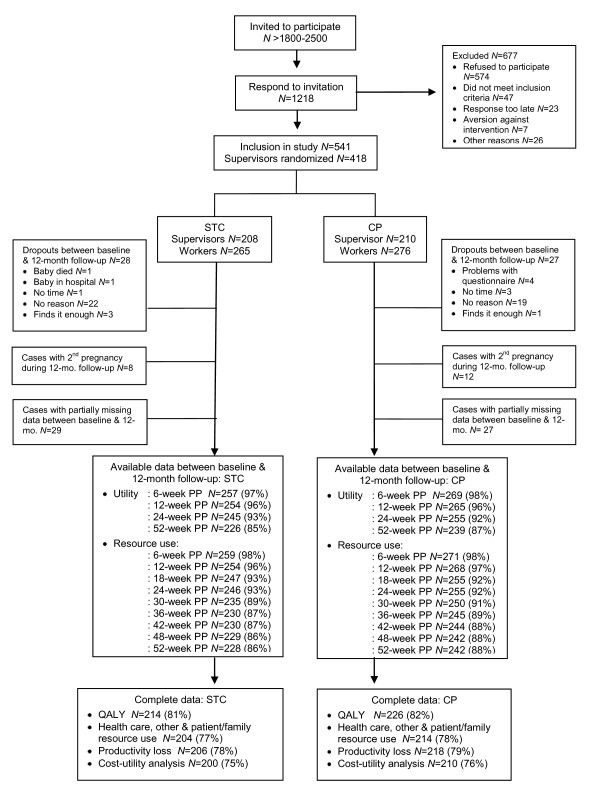
**Patient flow chart and data availability for Supervisor telephone contact (STC) and common practice (CP) between baseline and the 12-month follow-up**.

We invited 93 companies to participate in the study; 15 agreed. These 15 companies employed a range of 391 to 52,481 workers, and represented the health care (*N *= 9), service (*N *= 5) and government (*N *= 1) sectors of industry. Within these 15 companies, an estimated 1800 to 2500 working expectant mothers were invited to participate in the study. A total of 416 supervisors were randomized and 541 women participated in the study (figure 1). Baseline characteristics of the women in the two groups were similar (table 1).

**Table 1:** Baseline demographics, sick leave, health care use during first 6-weeks post-partum and utility for each group: Supervisor telephone contact (STC) and Common Practice (CP).

Patients baseline measures		STC (*N *= 265)	CP (*N *= 276)
Mean age in years (S.D.; range)		32 (4.0; 19-45)	32 (4.3; 19-42)

Marital status, *N *(%)	Single/Divorced	6 (2%)	8 (3%)
	Common law/Married	259 (98%)	268 (97%)

Level of education, *N *(%)	Low	25 (9%)	24 (9%)
	Intermediate	87 (33%)	96 (35%)
	High	153 (58%)	156 (57%)

1^st ^pregnancy, *N *(%)		126 (48%)	133 (48%)

Hours worked per week, *N *(%)	12-23	64 (24%)	61 (22%)
	24-35	122 (46%)	145 (53%)
	> 36	79 (30%)	70 (25%)

Predominant type of work, *N *(%)	Seated	94 (36%)	77 (28%)
	Standing	58 (22%)	71 (25%)
	Hand	109 (41%)	115 (42%)
	Heavy	4 (2%)	13 (5%)

Pre-partum sick leave hours, mean (S.D.)	1 year before pregnancy	69 (155) *	66 (147) *
	Pregnancy until start of maternity leave	112 (158) *	109 (178) *

Health care use, mean (S.D.)	General practitioner	0.3 (0.7) ^**†**^	0.3 (0.6) ^**†**^
	Midwife	0.2 (0.5) ^**†**^	0.3 (0.7) ^**†**^
	Gynaecologist	0.2 (0.5) ^**†**^	0.2 (0.5) ^**†**^
	Post-partum home care	40.0 (19.5) ^‡^	39.3 (19.2) ^‡^
	Partner	27.2 (43.3) ^‡^	28.3 (45.6) ^‡^
	Family/friends/volunteers	8.1 (21.4) ^‡^	11.7 (25.4) ^‡^

Utility, mean (S.D.; range)		0.92 (0.12; 0.31-1.0) ^¶^	0.91 (0.13; 0.17-1.0 ) ^¶^

* *N *for pre-partum sick leave based on company data: STC = 263; CP = 274; ^† ^Units of health care use are reported in the number of consultations. Available data at baseline for STC was *N *= 259 and CP was *N *= 271. ^‡ ^Units of health care use are reported in the number of hours. Available data at baseline for STC was *N *= 259 and CP was *N *= 271. ^¶ ^Available data at baseline for STC: *N *= 257 (97%) and CP: *N *= 269 (97%).

The response rates for both groups were similar at each measurement moment and ranged between 85% and 98%. Both groups had a similar number of women who had complete cost and QALY data: STC = 200 (75%); CP = 210 (76%). Baseline characteristics of the complete cases and women with second pregnancies were comparable between the groups. Except for a small, statistically significant between-group difference in the predominant type of work among women lost-to-follow-up, baseline characteristics of women lost-to-follow-up were similar. There was a small, statistically significant difference in education level between complete cases and those with incomplete data.

The proportion of women delivering at home versus in the hospital were comparable between the groups (STC: 88/259, 34%; CP: 84/259, 31%; Total: 172/530, 32.5%). There were no statistically significant between-group differences in the mean hours of parental leave (mean hours: STC = 114, S.D. = 158; CP = 120, S.D. = 177) nor proportion of women taking parental leave after the end of maternity leave (Proportion: STC = 91/206, 44%, CP = 99/218, 45%; mean hours: STC = 114, S.D. = 158; CP = 120, S.D. = 177).

### Resource use

Resource use per group and pooled data during the first 52-week post-partum period are presented in table 2. Rates of resource utilization were similar between the groups, except for the numbers visiting a psychologist (STC: 9/204, 4.4%; CP: 2/215, 0.9%; *p *= 0.03) and physical therapist (STC: 36/204, 17.6%; CP: 22/215, 10.5%; *p *= 0.03). There were no statistically significant between-group differences in terms of units of resource consumption.

**Table 2:** List of resources with respective price weights, observed resource use for each group during the pre- and post-intervention periods, and pooled values for the total study population during the first 52 week post-partum period.

			**Pre-intervention period (1**^**st **^6 weeks post-partum)^*^				Post-intervention follow-up (6-52 weeks post-partum)^*^				52-weeks post-partum^*^	
			
			STC		CP		STC		CP		Pooled total	
**HEALTH CARE SECTOR**	**Units**	**Price weight ** **(in **€**)**	**%**	**Mean (S.D.)**	**%**	**Mean (S.D.)**	**%**	**Mean (S.D.)**	**%**	**Mean (S.D.)**	**%**	**Mean (S.D)**
General practitioner	[No. of consults]	21.03 ^†^	23	0.3 (0.7)	23	0.3 (0.6)	49	1.2 (1.9)	53	1.1 (1.6)	57	1.5 (2.0)
	[No. of tel. consults]	10.51 ^†^	14	0.2 (0.6)	17	0.2 (0.5)	16	0.3 (0.7)	19	0.3 (0.8)	29	0.5 (1.0)
	[No. of house calls]	42.05 ^†^	8	0.1 (0.4)	12	0.2 (0.7)	3	<0.1 (0.2)	2	<0.1 (0.1)	13	0.2 (0.6)
Nurse practitioner	[No. of consults]	21.03 ^†^	1	<0.1 (0.1)	3	0.1 (0.4)	3	<0.1 (0.2)	4	0.1 (0.4)	6	0.1 (0.4)
	[No. of tel. consults]	10.51 ^†^	2	<0.1 (0.2)	3	<0.1 (0.3)	3	<0.1 (0.2)	1	<0.1 (0.2)	5	0.1 (0.3)
	[No. of house calls]	42.05 ^†^	3	0.1 (0.4)	3	<0.1 (0.3)	0	0 (0)	0	0 (0)	2	<0.1 (0.2)
Midwife	[No. of consults]	12.13 ^†^	17	0.2 (0.5)	19	0.3 (0.7)	6	0.1 (0.2)	9	0.1 (0.3)	23	0.3 (0.7)
	[No. of tel. consults]	6.06 ^†^	4	0.1 (0.3)	5	0.1 (0.3)	0	0 (0)	0.5	<0.1(0.1)	5	0.1 (0.4)
	[No. of house calls]	24.26 ^†^	25	0.7 (1.4)	23	0.7 (1.5)	0	0 (0)	1	<0.1 (0.1)	24	0.7 (1.5)
Obstetrican/Gynaecologist	[No. of consults]	58.29 ^†^	15	0.2 (0.5)	15	0.2 (0.5)	20	0.3 (0.8)	21	0.3 (0.8)	29	0.5 (1.0)
	[No. of tel. consults]	29.15 ^†^	2	<0.1 (0.1)	4	0.1 (0.3)	1	<0.1 (0.1)	1	<0.1 (0.1)	1	<0.1 (0.1)
Other Medical specialist	[No. of consults]	58.29 ^†^	2	<0.1 (0.3)	4	0.1 (0.4)	12	0.3 (1.0)	14	0.3 (0.8)	14	0.3 (1.1)
	[No. of tel. consults]	29.15 ^†^	1	<0.1 (0.9)	1	<0.1 (0.1)	4	0.1 (0.6)	2	0.1 (0.4)	4	0.1 (0.5)
Hospitalizations	[Length of stay in days]	350.80 ^†^	18	0.6 (1.7)	17	0.6 (1.8)	2	0.1 (0.5)	2	0.1 (0.5)	20	0.7 (1.8)
Uncomplicated delivery	[Length of stay in days]	...	8	2.7 (1.6)	6	2.4 (2.0)	...	...	...	...	6.9	0.2 (0.8)
OB-GYN-assisted	[Length of stay in days]	...	0	0 (0)	1	11 (1.4)	...	...	...	...	0.2	<0.1 (0.6)
C-Section	[Length of stay in days]	...	4	5.1 (1.3)	5	4.1 (1.2)	...	...	...	...	5.3	0.2 (1.1)
Peri-partum complications	[Length of stay in days]	...	6	3.4 (3.0)	6	3.4 (3.1)	<1	0.2 (1.1)	1	0.2 (1.1)	6.2	0.2 (1.0)
Other post-partum complications	[Length of stay in days]	...	0	0 (0)	0	0 (0)	1	<0.1 (0.4)	2	<0.1 (0.4)	1.7	0.1 (0.5)
Psychologist	[No. of sessions]	76.90 ^†^	0	0	<1	<0.1 (0.1)	4	0.2 (1.5)	1	0.05 (0.59)	3	0.1 (1.1)
Other psych specialists	[No. of sessions]	Variable ^‡, §^	1	<0.1 (0.1)	1	<0.1 (0.1)	2	0.1 (0.5)	2	0.1 (0.7)	3	0.1 (0.6)
Physical therapist	[No. of sessions]	23.68 ^†^	2	0.1 (0.5)	5	0.1 (0.9)	18	1.5 (4.6)	11	0.8 (3.1)	15	1.2 (4.1)
	[No. of fitness sessions]	13.20 ^‡^	0	0 (0)	0	0 (0)	1	<0.1 (0.4)	1	0.1 (0.7)	1	0.1 (0.5)
Manual therapist	[No. of sessions]	32.75 ^‡^	0	0 (0)	0	0 (0)	5	0.2 (0.8)	2	0.1 (0.9)	3	0.1 (0.9)
Exercise therapist-Mensendieck	[No. of sessions]	23.94 ^†^	1	<0.1 (0.2)	2	<0.1 (0.4)	3	0.1 (1.2)	3	0.4 (2.3)	3	0.3 (2.0)
	[No. of fitness sessions]	11.60 ^‡^	0	0 (0)	1	<0.1 (0.3)	0	0 (0)	0.5	0.2 (2.6)	<1	0.1 (2.0)
Other paramedical professionals	[No. of sessions]	Variable ^§^	0	0 (0)	1	<0.1 (0.4)	5	0.2 (0.9)	2	0.1 (1.2)	4	0.2 (1.1)
Medications	[% ]	Variable ^¶^	64		66			...		...	75	
Maternity aid	[Hours]	31.06 ^†^	97	40.0 (19.5)	98	39.2 (19.1)	...	...	...	...	98	39.6 (19.3)
Professional home/family care	[Hours]	Variable ^†^	2	0.4 (3.6)	2	0.5 (4.0)	0	0 (0)	0.5	0.1 (1.2)	2	0.4 (3.5)
**OTHER SECTOR**												
Occupational health physician	[No. of consults]	34.01 ^§^	<1	<0.1 (0.1)	0	0 (0)	8	0.2 (0.8)	7	0.1 (0.4)	7	0.2 (0.6)
	[No. of tel. consults]	11.34 ^§^	1	<0.1 (0.1)	1	<0.1 (0.1)	5	0.1 (0.5)	7	0.1 (0.5)	6	0.1 (0.5)
Occupational consultant/nurse	[No. of consults]	Variable ^§^	0	0 (0)	0	0 (0)	0.5	<0.1 (0.1)	0.5	<0.1 (0.1)	<1	<0.1 (0.1)
	[No. of tel. consults]	Variable ^§^	0	0 (0)	0	0 (0)	0.5	<0.1 (0.1))	1	<0.1 (0.1)	1	<0.1 (0.1)
Employer-covered fitness	[No. of sessions]	Variable ^§^	0	0 (0)	0	0 (0)	0	0 (0)	0.5	0.1 (2.1)	<1	0.1 (1.5)
All alternative care providers	[No. of sessions]	Variable ^§^	2	<0.1 (0.1)	2	<0.1 (0.1)	3	0.1 (0.8)	4	0.1 (0.8)	4	0.1 (0.8)
**PATIENT & FAMILY**												
Extra paid household help	[Hours]	8.64 ^†^	2	0.5 (6.5)	3	0.3 (1.9)	11	1.5 (6.4)	9	1.3 (6.2)	11	1.6 (6.7)
Extra day care	[Hours]	5.63 ^§^	1	0.1 (6.5)	1	0.2 (3.3)	3	0.5 (3.4)	2	0.2 (1.5)	3	0.5 (3.7)
Partner	[Hours]	8.64 ^†^	52	27.2 (43.3)	51	28.3 (45.6)	31	13.1 (32.3)	19	18.3 (59.1)	58	42.5 (74.2)
Family/friends/volunteers	[Hours]	8.64 ^†^	31		35	11.7 (25.4)	27	6.5 (21.6)	21	6.6 (22.2)	43	15.4 (33.5)
Sport or physical fitness activity	[%]	Variable ^§^	6	...	6	...	31	...	30	...	31	...
**PRODUCTIVITY LOSS**												
Sick leave from paid work	[Hours]	31.72 **	...	...	...	...	41	20.5 (60.6)	45	19.8 (49.0)	43	20.4 (55.4)
Work presenteeism	[Hours]	31.72 **	...	...	...	...	58	22.1 (35.2)	57	20.6 (29.8)	58	21.7 (32.7)
Total productivity loss	[Hours]	...	...	...	...	...	71	42.6 (76.2)	73	42.3 (64.6)	72	42.5 (70.3)

* Percent utilization and mean resource use in the pre-intervention period was based on available cases. Corresponding figures for during the post-intervention period were based on complete cases; The sample sizes for the STC and CP groups were 200 and 210, respectively. Pooled values were based on complete cost data: note that the sample sizes for the calculations varied because maternity aide care use was only measured during the first of 9 possible measurement moments, sick leave and work presenteeism during seven of 9 possible measurement moments and the remaining resource use at all 9 possible measurement moments during the 52-week follow-up. The sample size for maternity care aide use was *N *= 527, for sick leave from paid work and work presenteeism *N *= 424, and for the remaining types of resource use *N *= 418.

Price weight sources: ^† ^Dutch guidelines for costing studies; ^‡^Dutch Central Organization for Health Care Charges (CTG); ^§ ^Respective providers or professional organizations; ^¶ ^Royal Dutch Society for Pharmacy; ** Weighted average for women aged 19-45 years calculated from the Dutch guidelines for costing studies.

During the first 52-weeks post-partum, the top five most commonly used resources were: 1. maternity aid care (98%); 2. medications or assistive devices (75%); 3. informal care by partner (58%); 4. GP consults (57%); and 5. work presenteeism (46%). Less than 10% of the women used occupational health services.

The top five mean volumes of consumed resources were: 1. informal care by partner (42.5 hours); 2. maternity aid care (39.6 hours); 3. productivity loss due to work presenteeism (21.7 hours); 4. productivity loss due to sick leave (20.4 hours); and 5. informal care by family/friends/volunteers (15.4 hours).

### Costs, effects and cost-utility analysis

At the end of the follow-up period, there were no statistically significant between-group differences in QALYs (STC mean = 0.928, S.D. = 0.094; CP mean = 0.935, S.D.= 0.087; mean difference = -0.007, 95% CI: -0.023; 0.009). Also, the groups did not differ in terms of mean number of sick leave hours (STC = 26.1, S.D. = 66.3; CP = 24.6, S.D. = 65.2; mean difference = 1.5, 95% CI: -10.1; 13.0), work presenteeism hours (STC = 24.1, S.D. = 36.7; CP = 20.7, S.D. = 29.8; mean difference = 3.4, 95% CI: -2.2; 9.1) or total productivity loss hours (STC = 50.2, S.D. = 84.2; CP = 45.3, S.D. = 77.6; mean difference = 4.9, 95% CI: -9.1; 18.9).

Mean costs per group, mean differences and pooled costs are presented in table 3. Health care sector costs represent medical costs covered under basic health insurance for all Dutch citizens. Other sector costs represent medical costs covered by companies or supplemental insurance. Patient and family costs represent non-medical costs paid out-of-pocket. Productivity loss costs represent costs due to sick leave and work-presenteeism. There were no statistically significant between-group cost differences for any of the component or total costs at the end of follow-up. The STC did not result in any cost savings in terms of reduced sick leave or work presenteeism compared to common practice. Taken together, health care sector costs accounted for 46.4% of the total costs, productivity loss costs for 36.8%, patient/family costs for 16.5% and other sector costs for 0.3%. Of the total productivity loss costs, 48% was attributable to sick leave and 52% to work presenteeism.

**Table 3:** Respective group mean costs and mean cost differences during the post-intervention period (from 6-52 weeks post-partum), and pooled totals for the first 52-weeks post-partum (including 1^st ^6-weeks post-partum) based on imputed data and reported in 2006 Euros.

Components	STC*	CP*	Mean difference	**Pooled**^**†**^
			
	Mean (S.D.)	Mean (S.D.)	(95% CI)	Mean (95% CI)
Health care sector	199 (375)	169 (326)	30 (-32; 99)	1707 (1613; 1765)
Other sectors	11 (39)	10 (51)	1 (-8; 9)	11 (8; 16)
Patient/family	273 (603)	274 (616)	-1 (-121; 127)	608 (528; 690)
Sick leave	662 (1682)	625 (1655)	37 (-252; 334)	643 (506; 821)
Work presenteeism	765 (1164)	655 (944)	109 (-66; 298)	709 (615; 804)
Total productivity loss	1427 (2297)	1281 (2084)	146 (-228; 528)	1352 (1158; 1561)
Total costs	1911 (2867)	1734 (2644)	177 (-293; 678)	3678 (3386; 3951)

* STC = Supervisor telephone contact; CP = Common Practice.

^**† **^Pooled mean costs for health care sector and patient/family are higher than the mean group values during follow-up because the pooled means include costs during the first 6-weeks post-partum. During the first 6-weeks post-partum (i.e. pre-intervention phase), health care resource use and informal care by partner and family were high and similar between groups.

**Figure 2:**
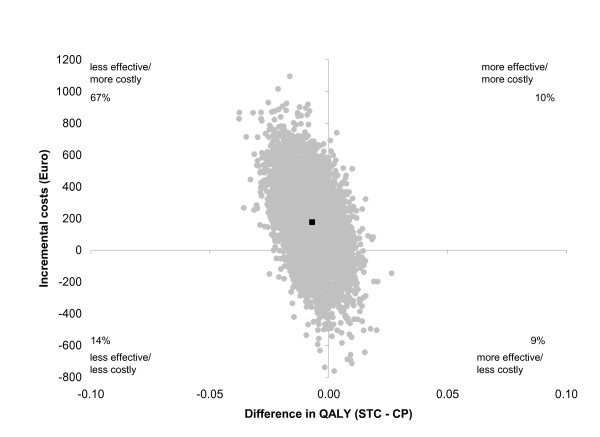
**Cost-utility plane illustrating the distribution of the joint cost-QALY pairs from the main analysis and the mean ICUR ( - 25,440)**.

The joint cost-QALY pairs were distributed around all four quadrants of the cost-utility plane. The majority of the joint cost-QALY pairs and the mean ICUR were located in the northwest quadrant indicating that the intervention was less effective and more costly (figure 2). For willingness-to-pay levels from €0 through €50,000, there was a 20% chance of the STC intervention being more cost-effective than common practice (cost-effective acceptability curve not shown). Findings of all three sensitivity analyses were in the same direction as the main analysis (table 4).

**Table 4:** Mean cost differences (ΔC), mean effect differences (ΔE), incremental cost-utility ratios and distribution of the joint cost-effect pairs in the cost-utility plane from the main cost-utility analysis and corresponding three sensitivity analyses (SA1, SA2 and SA3).

Analysis^*^	Sample size		ΔC (95% CI)	ΔE (95% CI)	ICUR	Distribution in CU-plane			
					
	STC	CP				NE^†^	SE^‡^	SW^§^	NW^¶^
Main	260	272	177 (-293; 678)	-0.007 (-0.023; 0.009)	-25,440 **	10%	9%	14%	67%
SA1	260	272	186 (-350; 751)	-0.007 (-0.023; 0.009)	-26,774 **	10%	9%	16%	65%
SA2	252	260	-194 (-295; 751)	-0.005 (-0.021; 0.117)	-42,569 **	17%	13%	10%	59%
SA3	200	210	41 (-404; 487)	0.001 (-0.017; 0.015)	-40,939 **	20%	25%	17%	38%

* In the main analysis, ΔE = mean difference in QALY, ΔC = mean difference in total costs in which the productivity loss costs were estimated by the FCM; in SA1, ΔC = mean difference in total costs in which the productivity loss costs were estimated by the HCA; SA2 is a repetition of the main analysis in which women who became pregnant a second time during follow-up (*N *= 20) were excluded; SA3 is a repetition of the main analysis using only the complete cases.

^† ^Refers to the northeast quadrant of the CU-plane, which indicates that STC is more effective and more costly than CP.

^‡ ^Refers to the southeast quadrant of the CU-plane, which indicates that STC is more effective and less costly than CP.

^§ ^Refers to the southwest quadrant of the CU-plane, which indicates that STC is less effective and less costly than CP.

^¶ ^Refers to the northwest quadrant of the CU-plane, which indicates that STC is less effective and more costly than CP.

** All of these negative incremental cost-utilty ratios (ICURs) were located in the NW quadrant, along with the majority of joint cost-effect pairs, indicating that the STC was less effective and more costly than CP.

## Discussion

In this study, we evaluated the cost-utility of a supervisor telephone contact intervention during maternity leave with common practice among working mothers. The results demonstrate that the intervention was neither clinically nor economically superior to common practice. Therefore, implementation is not indicated. We pooled the data of the two groups to gain insight into the resource use during the first year post-partum. The proportion of study population giving birth in the hospital (67.5%) or at home (32.5%) was comparable to 2005-2007 national figures, 70% and 29%, respectively [30]. The utilization results suggest that access to health care and use of services following childbirth is good. Nearly 100% of the women received help from a maternity aid. Also, informal care by partners, family, friends and volunteers plays a considerable role. Work presenteeism accounted for 50% of the total productivity loss.

### Strengths & limitations

A key strength of the study is that it is the first to investigate the cost-utility of interventions for post-partum occupational health problems. This also underscores the need for further research to identify cost-effective ways to prevent sick leave and improve quality of life in working mothers following childbirth. Additional strengths include the use of a randomized controlled trial design, with randomization at the supervisor-level to prevent contamination; and a good follow-up rate in which only 10% were lost-to-follow-up.

Main limitations are risks for selection bias, limited generalizability and the logic of the intervention. With regards to selection bias, there are two considerations. First, we had difficulty recruiting companies to participate in this study. Participation may be reflective of the degree of problem recognition within the company, and in turn be reflective of the current workplace culture, policies for maternal health issues, workers' health and sick leave. As such, the participating companies may represent an optimal setting and any observed effect could be an overestimation. However, we did not observe an effect, therefore, selection bias in this sense is not likely. Second, the women in the study had higher levels of education compared to the general Dutch female population. Higher levels of education are associated with higher socioeconomic status, and better post-partum health outcomes have been reported for such groups of women [31]. It is unclear if the intervention would have been more effective in a population with a lower level of education.

The generalizability of the results may be limited by differences in social context between The Netherlands and other countries. For example, the length of maternity leave in The Netherlands is 16 weeks with full pay, whereas mothers in the UK are entitled to 52 weeks of paid and unpaid leave [32]. In the USA, paid maternity leave is not standard: among the best U.S. employers, 7% did not offer any paid leave, 17% between 1-4 weeks, 48% between 5-8 weeks, 20% 9-12 weeks and 8% more than 12-weeks [33]. Also, differences in the organization of primary and occupational health care, and the older average age of Dutch women giving birth may result in different patterns of resource use [34].

Other differences may be related to labour participation. In general, the characteristics of Dutch female labour participation is comparable to many countries. The Dutch female participation rate (59.2%) is slightly higher than the world average (51.9%) but similar to, for example, Sweden, Canada, United States and Australia. The Dutch rate is lower than the rates in, for example, Iceland, Rwanda, Kenya and Cambodia (>70%), and it is higher than the rates in, for example, Belgium, Italy, Japan and Turkey (< 50%) [35]. The percentage of Dutch women working in non-agricultural sectors (45.7%) is higher than the world average (36.9%), however, in line with countries around the world such as the United States, Canada, Belgium, Norway, Thailand, Argentina and the Central African Republic [36]. The majority of the participants in our study worked in the health care sector, which worldwide, traditionally has a predominantly female workforce.

The intervention was based on the logic that early identification of clinical post-partum morbidities by the supervisor telephone contact would lead to timely involvement of occupational health to identify potential barriers and corresponding solutions for returning to work given the clinical post-partum morbidity. This logic behind linking clinical post-partum morbidities with occupational health is that occupational health has a key role in maintaining a link to the workplace, which facilitates return-to-work. The one-time phone call served as a starting point for preventative actions that could be initiated in the workplace by the occupational health physician in order to optimalize return-to-work.

### Methodological considerations

Reasons for the lack of effect may be poor conceptualization of the intervention, low intensity of the intervention, use of parental leave, and characteristics of the study population. First, poor conceptualization is unlikely. Recently, Kant et al. reported that structured early consultation with the occupational physician reduced sick leave in a high risk population of office workers [37]. Earlier involvement of occupational physicians was the aim of the intervention.

Second, it is possible that intensity of the intervention was too low. The one-time phone call at 6-weeks may have been insufficient as new problems may arise between 6-weeks and the official end of the maternity leave. Follow-up contact, for example, at 8 or 9-weeks or at biweekly intervals until expected return-to-work, may be improvements.

Third, in The Netherlands, all working mothers are eligible to take complete or partial parental leave following maternity leave. It is possible that parental leave may mask sick leave. However, the numbers postponing return-to-work after maternity leave by using complete parental leave were comparable between the groups (STC = 17%; CP = 20%). Also, given that the women were eligible for sick leave benefits up to 2-years, we do not think the women who took complete parental leave did so as a substitute for sick leave. However, the use of partial parental leave may help women make the transition from maternity leave to return-to-work as usual and as such, have a preventative effect on sick leave or work presenteeism. Proportions of women in both groups taking parental leave at 18-, 24- and 52-weeks post-partum were comparable: STC = 21%, 25% and 18%; CP = 24%, 23% and 15%.

Last, the most probable reason for lack of effect likely relates to the characteristics of the study population. The women in this study represented a healthy group of working mothers: utilities of the women were above 0.9 at each measurement moment and health care consumption throughout the follow-up period was low. Furthermore, the sick leave rates of the study population were lower than those reported in the literature used to conceptualize the intervention. In our study, only 2% of the women took sick leave at the end of their maternity leave versus 29%. This unexpected result suggests that there may not have been a problem upon which to intervene.

With regards to the cost description, we excluded parental leave. However, parental leave may be considered another form of productivity loss during the first 52-weeks post-partum. In our study, 45% of the subjects took either complete or partial parental leave, with the mean number of parental leave hours taken in the 52-weeks post-partum being 117 (S.D. = 168). If parental leave is considered in the total costs, then parental leave would represent the main cost driver and account for 52% of the total costs.

We conducted our economic evaluation from a societal perspective. However, additional analyses from a specific stakeholder perspective can be informative and desirable. In The Netherlands, the company is a key stakeholder as it has the responsibility to pay for interventions as well as an opportunity to gain in terms of decreased sick leave and work presenteeism. With respect to this study, a cost analysis from a company's perspective would compare the interventions costs (i.e. other sector costs consisting of occupational health and supervisor time) to the costs of productivity loss (i.e. all work presenteeism and sick leave not subsequent to maternity leave) between the groups. The mean intervention costs were €1 higher in the STC group as were the mean work presenteeism costs (€109; see Table 3). Estimation of mean group differences in sick leave costs limited to sick leave not subsequent to maternity leave was also higher in the STC group (€89; 95% CI: -192; 379). Therefore, from a company's perspective, implementation of STC is not warranted.

In occupational health care, the scope of productivity loss has traditionally focussed solely on sick leave. In recent years, decreased productivity while at work, i.e. work presenteeism has received more attention. However, little is known about work presenteeism following childbirth. In this study, 58% of the women reported work presenteeism and the contribution of sick leave and work presenteeism to total productivity loss hours was roughly 50:50. Our finding that work presenteeism was a common problem is in line with other studies investigating other working populations and health problems. For example, Aronsson et al. [8] found that 37% of the Swedish workforce experienced work presenteeism. Among workers with high physical load jobs and health problems, 50% reported work presenteeism [38]. That work presenteeism can represent a considerable proportion of total productivity loss, is in line with findings from Burton et al. in which the ratio of sick leave:work presenteeism was 40:60 [39]. As such, future studies on return-to-work following childbirth should include work presenteeism.

## Conclusions

In this study, very few women had post-partum health problems that acted as barriers to return-to-work. In this healthy population, early supervisor telephone contact during maternity leave was not cost-effective compared to common practice and widespread implementation is not warranted. The cost-utility of early supervisor telephone contact should be evaluated in a high-risk population and studied in other socio-political systems. The lack of comparable studies underscores that return-to-work following childbirth is under-researched. Traditionally, the focus of return-to-work interventions has been on sick leave. Work presenteeism may also form a substantial portion of total productivity loss and should be considered in future research.

## List of Abbreviations

ABC bootstrapping: Approximated bias-corrected bootstrapping; CU-plane: cost-utility plane; CP: common practice; CUA: cost-utility analysis; ΔC: difference in mean costs; ΔE: difference in mean effects; FCM: Friction Cost Method; HCA: Human Capital Approach; HPQ: Health and work Performance Questionnaire; ICUR: incremental cost-utility ratio; MI: multiple imputation; MICE: Multivariate Imputation by Chained Equations; *N*: sample size; PP: post-partum; QALYs: Quality-adjusted life years; RTW: return-to-work; SA: sensitivity analysis; STC: supervisor telephone contact; S.D.: standard deviation; TC_FCM_: total costs in which productivity loss costs were estimated using the friction cost method; UK: United Kingdom; US/USA: United States/United States of America; 95% CI: 95% confidence interval.

## Competing interests

This study was funded internally by the Body@Work Research Center, Amsterdam, The Netherlands, and not by any external funding agencies. The authors declare that they do not have any competing interests.

## Authors' contributions

KU participated in the design of the economic evaluation and data collection, conducted the data analysis and drafted the manuscript. SGMS was responsible for the data collection, participated in the overall design of the trial and research protocol. MCdB participated in the design of the economic evaluation and advised in the data analysis. MNMvP originated the idea for the study and participated in the overall design of the trial and research protocol. MWH was involved in the data analysis. WvM participated in the design of the trial and research protocol. MvT participated in the design of the economic evaluation and advised in the data analysis. All authors provided feedback on earlier drafts, and have read and approved the final version of the manuscript.

## Pre-publication history

The pre-publication history for this paper can be accessed here:

http://www.biomedcentral.com/1471-2458/11/57/prepub
